# Organelle‐Targeting Nanoparticles

**DOI:** 10.1002/advs.202411720

**Published:** 2025-01-13

**Authors:** John Soukar, Nicholas A. Peppas, Akhilesh K. Gaharwar

**Affiliations:** ^1^ Interdisiplinary program in Genetics and Genomics Texas A&M University College Station TX 77843 USA; ^2^ Department of Biomedical Engineering College of Engineering Texas A&M University College Station TX 77843 USA; ^3^ Department of Biomedical Engineering University of Texas at Austin Austin TX 78712 USA; ^4^ Institute of Biomaterials Drug Delivery and Regenerative Medicine University of Texas at Austin Austin TX 78712 USA; ^5^ Department of Chemical Engineering University of Texas at Austin Austin TX 78712 USA; ^6^ Department of Surgery and Perioperative Care Dell Medical School University of Texas at Austin Austin TX 78712 USA; ^7^ Department of Pediatrics Dell Medical School University of Texas at Austin Austin TX 78712 USA; ^8^ Department of Material Science and Engineering College of Engineering Texas A&M University College Station TX 77843 USA

**Keywords:** endoplasmic reticulum, endosome, Golgi apparatus, mitochondria, nanomaterials, nucleus, targeted delivery

## Abstract

Organelles are specialized subunits within cells which carry out vital functions crucial to cellular survival and form a tightly regulated network. Dysfunctions in any of these organelles are linked to numerous diseases impacting virtually every organ system in the human body. Targeted delivery of therapeutics to specific organelles within the cell holds great promise for overcoming challenging diseases and improving treatment outcomes through the minimization of therapeutic dosage and off‐target effects. Nanoparticles are versatile and effective tools for therapeutic delivery to specific organelles. Nanoparticles offer several advantageous characteristics, including a high surface area‐to‐volume ratio for efficient therapeutic loading and the ability to attach targeting moieties (tethers) that enhance delivery. The choice of nanoparticle shape, size, composition, surface properties, and targeting ligands depends on the desired target organelle and therapeutic effect. Various nanoparticle platforms have been explored for organelle targeting, such as liposomes, polymeric nanoparticles, dendrimers, and inorganic nanoparticles. In this review, current and emerging approaches to nanoparticle design are examined in the context of various diseases linked to organelle dysfunction. Specifically, advances in nanoparticle therapies targeting organelles such as the nucleus, mitochondria, lysosomes/endosomes, Golgi apparatus, and endoplasmic reticulum are comprehensively and critically discussed.

## Graphical abstract

This review examines nanoparticle‐based therapies targeting organelles like the nucleus, mitochondria, and lysosomes. Nanoparticle design, targeting strategies, and their applications in treating diseases linked to organelle dysfunction are discussed, highlighting the potential for improved treatment outcomes and reduced off‐target effects.

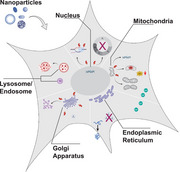

## 1 Introduction

Cellular organelles, namely the nucleus, mitochondria, lysosomes/endosomes, Golgi apparatus, and endoplasmic reticulum regulate a host of biological processes including protein synthesis, energy production, waste disposal, signal transduction, and cell growth. Dysfunctions in any of these organelles are linked to numerous diseases impacting virtually every organ system in the human body.^[^ ^1^, ^2^, ^3^, ^4^, ^5^, ^6^ ^]^ A range of diseases such as cancer, diabetes, arthritis, Alzheimer's disease, and cardiomyopathy have been linked to the dysfunction of organelles, typically stemming from genetic defects as well as environmental factors. Thus, the targeted delivery of therapeutics to organelles holds significant potential for overcoming some of these difficult‐to‐treat diseases.^[^ ^7^, ^8^, ^9^, ^10^ ^]^ The precise delivery of therapeutics to individual organelle types can lead to improved outcomes through optimized dosage requirements, decreased toxicity, and minimized off‐target effects.

Nanoparticles have emerged as a versatile and effective tool for targeted therapies due to their small size, high surface area, and the ability to carry a range of therapeutics biomolecules.^[^ ^7^, ^10^, ^11^ ^]^ Their small size and modular surface chemistry enable nanoparticles to overcome biological barriers and facilitate targeted delivery to specific cells, tissues, and organs.^[^ ^12^, ^13^ ^]^ A range of therapeutic drugs, macromolecules, and biologics can be encapsulated within the interior or loaded on the surface of nanoparticles via physical adsorption, electrostatic or ionic interactions, and/or covalent conjugation. The diverse range of interactions between therapeutics and nanoparticles facilitates tailored release of loaded therapeutics including burst, sustained, and/or on‐demand delivery.^[^ ^14^ ^]^

Nanoparticles can be administered through two primary routes: systemic or localized delivery. Regardless of administration, once they successfully navigate the variety of biological barriers, they arrive at their intended target organs, or tissue. Within the extracellular environment, nanoparticles can interact with proteins as well as cells, and are often internalized through one of several endocytosis mechanisms, mediated by surface receptors.^[^ ^7^, ^15^, ^16^, ^17^ ^]^ Upon entering the cytoplasm, the therapeutic‐loaded nanoparticles may accurately navigate to specific subcellular compartments while evading degradation. The strategy of sub‐cellular targeting holds considerable promise for enhancing clinical outcomes and minimizing side effects associated with traditional systemic administration of therapeutics. This review aims to provide an in‐depth discussion on the inherent challenges in organelle‐specific targeting, as well as how recent advancements in nanomedicine are providing solutions. We will also delve into the specific roles that key organelles play in the pathology of various diseases and present nanoparticle approaches to boost organelle functions. We will focus on the mitochondria, nucleus, lysosomes, Golgi apparatus, and endoplasmic reticulum as the primary targets of interest. Finally, we will spotlight some of the most promising emerging techniques in the realm of organelle‐specific targeting.

## 2 Role of Organelles in Disease

Within the eukaryotic cell, organelles serve as specialized compartments, each dedicated to carrying out designated processes. This distinctive compartmentalization allows for a division of labor within the cell, contributing to its overall efficiency and functionality. For example, the mitochondria are responsible for energy production, the nucleus controls genetic information and directs cell activities, the endoplasmic reticulum is involved in protein and lipid synthesis, and the lysosomes handle waste disposal. This functional segregation ensures that complex biochemical processes can occur simultaneously without interference, thereby enhancing the cell's ability to adapt and respond to its environment.

Even as organelles serve as discrete compartments of biological processes, they are inseparably intertwined. Each organelle, responsible for the generation, maintenance, and processing of a host of biological molecules, form a network that makes up cellular homeostasis.^[^ ^18^, ^19^, ^20^ ^]^ This is maintained by organelle cooperation and contact, with rapid material and information exchange and execution of various biological processes and related cellular behaviors such as autophagy, inflammation, and apoptosis. The balance is delicate, and dysfunction can affect other organelles and change or destroy the physiological and biochemical activities and structure of cells, leading to the occurrence and development of diseases.^[^ ^21^, ^22^ ^]^

**Figure 1:**
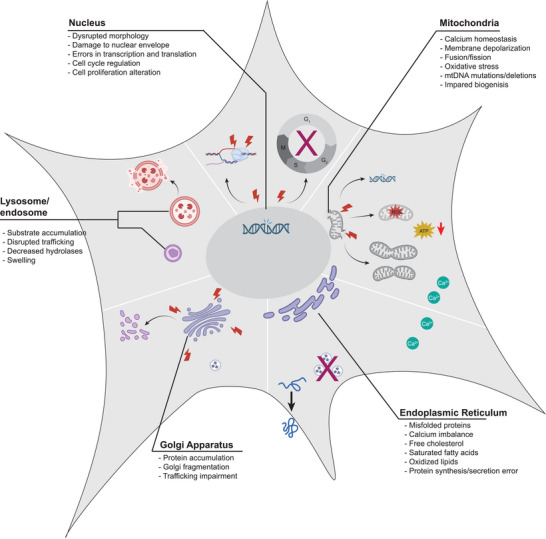
Organelles of the cell and their associated dysfunctions. Each organelle of the cell is responsible for a niche set of functions that maintain homeostasis and cellular health. Biology utilizes this organized system, each with emergent properties, to maintain a complex set of functions critical for survival. Disruptions in these tightly controlled processes range in a variety of potential manifestations specific to the role the organelle plays for the cell. The figure was created using icons from BioRender.com and modified and finalized in Adobe Illustrator.

Dysfunctions in specific organelles are at the root of many diseases (**Table** **1**). Far from being static structures, organelles form a dynamic cellular network, constantly exchanging information to coordinate responses to stress. These responses can involve swift action by the endoplasmic reticulum (ER) to prioritize cell survival through regulation of ER chaperones and proteins of the degradation system and increased ATP generation by the mitochondria—fueled by metabolites produced through lysosomal degradation processes. Given this close interconnection, a single organelle's dysfunction can trigger a domino effect of complications, affecting not only the cell itself but also specific tissues and entire organ systems. Consequently, diseases affecting nearly every organ in the body can be traced back to a specific organelle's dysfunction (**Figure** **1**).

**Table 1:** List of diseases, along with their associated organelle dysfunctions.

Organelle	Dysfunction type	Condition	Refs.
Endosome/ Lysosome	Lysosomal Storage Disorders	Pompe, Tay Sachs, Sandhoff, Gaucher, Krabbe	[4, 54, 168, 169]
	Loss of Function	Rheumatoid arthritis, Huntington's, Parkinson's	[2, 54, 170, 171]
	Alterations to transport	Amyotrophic lateral sclerosis (ALS)	[54]
	Unbalanced pH Control	Alzheimer's, Multiple sclerosis, Crohn's	[2, 6, 52, 54, 170, 172]
Mitochondria	Loss of Function	Aging, Ischemic Stroke	[96]
	Dysfunction of Complex I	Leber's hereditary optic neuropathy, Parkinson's	[91, 94, 99]
	mtDNA deletions/rearrangements	Hearing loss, Myoclonic epilepsy, Stroke and seizures, Lactic acidosis, Endocrine disorders, Adult‐onset diabetes, Atherosclerosis, Cancer	[13, 88, 100]
	Errors in transcription	Huntington's	[94]
	Permeability	Alzheimer's	[94]
	Dysfunction of Complex V	Leigh syndrome	[13]
	Oxidative Phosphorylation Deficiency	Cardiomyopathy	[13]
	Dysregulation	Cancer	[95]
Endoplasmic Reticulum	ER‐endosome trafficking	Parkinson's	[129, 134, 173]
	ER‐organelle trafficking	ALS, Alzheimer's, Parkinson's, Epileptic encephalopathy	[129, 134, 173]
	Mutant Protein Storage	Cerebral hypoxia, Nonalcoholic fatty liver disease, ALS, Parkinson's, Alzheimer's, Cardiovascular disease	[128, 129, 134]
	Increased demand	Type 2 Diabetes, Cancer	[129, 134, 145]
Golgi Apparatus	ER to Golgi Defects	Rhabdomyolysis, Early‐onset neurodevelopmental disorders, Microcephaly, Epilepsy, Ataxia, ALS	[130, 150]
	Golgi to Membrane Defects	Spinal muscle atrophy, Pettigrew syndrome, Menkes syndrome	[130, 150]
	Dysregulation	Cancer	[150]
Nucleus	Nuclear pore dysfunction	Huntington's, Multiple Sclerosis, ALS, Parkinson's, Alzheimer's	[3, 73, 78]
	Integrity loss	Emery‐Dreifuss muscular dystrophy, Dilated cardiomyopathy, Limb‐girdle muscular dystrophy	[3, 72]

Understanding the roles of organelles in disease provides essential context for the conscious design of targeted therapies, such as those utilizing nanoparticles. The dysfunction or altered state of specific organelles—such as mitochondria in neurodegenerative diseases or the endoplasmic reticulum in cancer—underscores the importance of precise targeting. Nanoparticles offer an ideal platform for subcellular delivery, but their efficiency of uptake depends heavily on interactions with biological membranes and components, which can be further altered in these organelle‐driven pathologies. Therefore, nanoparticles must overcome these cellular barriers to reach the desired organelle and ensure that the therapeutic payload reaches its intended destination.

## 3 Nanoparticle Uptake Mechanism

**Figure 2:**
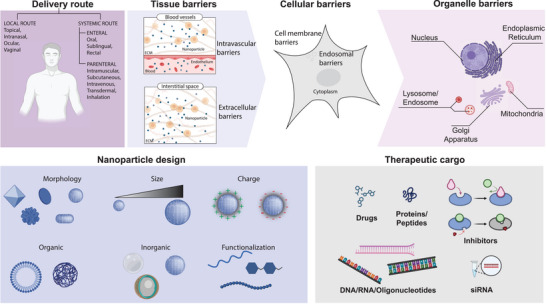
Different delivery routes of therapeutics. The bioavailability of therapeutics decreases progressively from the tissue level to the cellular level and further to the organelle level, resulting in only a small fraction reaching the target organelles. To overcome this issue, various nanoparticle designs can be employed for subcellular localization. Nanoparticle characteristics—including morphology, size, shape, charge, and functionality—significantly affect the transport and delivery of therapeutics. A range of therapeutics, such as small molecules, nucleic acids, and proteins, can be delivered using these nanoparticles. The figure was created using icons from BioRender.com and modified and finalized in Adobe Illustrator.

The first barrier to drug delivery is the route of administration. In vitro, this is of limited concern. However, in vivo, nanoparticles encounter multiple barriers across different biological levels (**Figure** **2**). Upon injection, nanomaterials interact with the bloodstream and often accumulate in the liver and kidneys, limiting the amount that reaches the intended organ or tissue—a process that is not guaranteed.^[^ ^23^, ^24^ ^]^ Once nanoparticles reach the target tissue, they must navigate the extracellular matrix and overcome interstitial pressure to penetrate and distribute effectively within the tissue environment. At the cellular level, nanoparticles face the challenge of crossing the cell membrane, which can vary in permeability depending on the cell type and its physiological state. Even after cellular entry, nanoparticles must contend with intracellular barriers, such as avoiding endosomal entrapment and ensuring delivery to specific organelles. Targeting dysfunctional organelles adds another layer of complexity, as it requires precise interaction with subcellular structures without disrupting normal cellular functions. To overcome these barriers and improve targeting, researchers utilize specific characteristics unique to pathological tissues and develop localized administration strategies. The ability to target pathological tissues has been extensively explored and reported.^[^ ^25^ ^]^ This review focuses on the events that occur once nanoparticles reach the tissue sites and explore strategies to further enhance specificity to dysfunctional organelles.

Successful intracellular delivery can be done either passively or actively. The reliance of passive targeting on physicochemical properties makes it difficult to target specific cell types in a mixed population. Such targeting is used for in vitro investigations. Nanostructures with a positive surface charge bind with the cell membrane and induce cell uptake. This is achieved using cationic molecule‐containing monomers (such as ammonium or other groups containing structures that can be ionized, such as amines.^[^ ^16^, ^26^ ^]^ The positive charge causes electrostatic attraction between the carrier and negatively charged cell surface proteoglycans. This approach also comes with potentially significant cytotoxicity and does not guarantee cytosolic delivery. Bio‐specific approaches achieve intracellular uptake by functionalizing the surface of nanodevices with naturally or synthetically derived cell surface receptor ligands such as the HIV tat peptide, the RGD sequence, epidermal growth factor, or transferrin.^[^ ^26^, ^27^, ^28^, ^29^, ^30^, ^31^ ^]^

The cellular internalization, or endocytosis, of nanoparticles is a complex and tightly controlled process regulated by the plasma membrane. The semi‐permeable, negatively charged, plasma membrane is the primary barrier that protects the cell from the outside environment. The primary endocytosis mechanisms include phagocytosis, clathrin‐mediated endocytosis, caveolin‐mediated endocytosis, clathrin/caveolae‐independent endocytosis, and macro‐pinocytosis. These endocytotic pathways have been previously studied extensively and are dictated by physiochemical characteristics of the nanoparticles.^[^ ^15^, ^32^ ^]^ Each of these pathways presents certain challenges for nanoparticle uptake as detailed in **Table** **2**. In summary, the size, shape, stiffness, and surface chemistry of nanoparticles interact with the elasticity of the cell membrane and present surface receptors. During phagocytosis and micropinocytosis, membrane wrapping induces significant forces on the cell where the membrane bends away from its natural state, utilizing membrane reservoirs increasing tension; when specific receptors are called, nanoparticles docks onto the cell membrane, ligand–receptor bindings introduce a time delay through diffusion of the receptors to the binding sites,^[^ ^17^ ^]^ thereby setting a characteristic time scale of endocytosis. Not only does endocytosis affect the cell but exerts forces on the particles as well; for example, soft nanoparticles see significant shape changes during membrane wrapping.^[^ ^17^ ^]^

**Table 2:** Cellular uptake pathways for nanoparticles.

Pathway	Important considerations	Nanoparticle properties
Direct Translocation	Very limited in scope	Functions for highly charged particles
Clathrin Mediated	Present in all cells	Large particles up to 100 nm in size, common for spherical nanoparticles, rigid
Caveolin Mediated	Not Present in all cells	Size range of 50–80 nm, avoids lysosome; Generally not common for nanoparticles
Clathrin/Caveolin Independent	Not present in all cells	Common of PEG or PLGA modified, tubular nanoparticles
Phagocytosis	Many non‐professional phagocytic cells simply lack receptors but have machinery	Very large particles upward on 200 nm, hydrophobic surfaces, not common for pegylated nanoparticles due to hydrophilicity
Macropinocytosis	Can be stimulated via growth factors	Very large particles upward of 200 nm up to 1 um in size, common for positively charged particles, flexible

Factors such as presence of oxygen and higher temperatures can also influence nanoparticle endocytosis. The specific effects of environmental temperature and oxygen on the quantity of nanoparticle uptake are beyond the scope of this review; however, it is established that low‐temperature environments result in a decreased interaction of particles with proteins and cellular membranes, rapidly increasing at physiological temperatures.^[^ ^15^, ^33^, ^34^ ^]^ It is common to synchronize internalization and binding by initially incubating nanoparticles with cells at 4 °C to inhibit uptake. Unbound particles are then washed away and the cells return to 37 °C to initiate uptake. Further, hypoxic environments, such as those found within cancer cells, have been found to inhibit endocytosis pathways but can harnessed as a potential therapeutic target in some nanoparticles.^[^ ^35^, ^36^ ^]^

Upon endocytosis, the physiochemical characteristics of nanoparticles dictate their intercellular fate. Extensive research has been conducted on various nanoparticles, elucidating their potential in surmounting barriers to intracellular entry and localization.^[^ ^37^ ^]^ Organic nanoparticles are fabricated from proteins, carbohydrates, lipids, or other organic compounds. These encompass peptide‐based nanoparticles, derived from surfactant‐like peptides or polypeptides including viral structural proteins or capsids; lipid‐based nanoparticles, consisting of natural or synthetic phospholipids, lipids, liposomes, or emulsions employed for delivering therapeutic agents;^[^ ^38^, ^39^, ^40^ ^]^ and polymer‐based nanoparticles composed of synthetic and natural polymers such as poly‐L‐lysine, poly‐ethylenimine, and others. Conversely, inorganic nanoparticles show distinct chemical and physical properties compared to their bulk counterparts, including optical, magnetic, thermodynamic, electrochemical, and catalytic properties.^[^ ^41^ ^]^

Common inorganic nanoparticles comprise metal, silica, carbon‐based, and metal‐oxide nanoparticles.^[^ ^42^ ^]^ The intercellular fate of each of these nanoparticles is influenced by several factors. Key properties of nanoparticles such as size, shape, and charge dictated their stability in the blood, interaction with proteins and cells, as well as intracellular trafficking. Further, the rational design of synthetic polymers is equally attractive because of their facile manufacture, large carrying capacity, tunable physicochemical characteristics, and modulation of biological activity through attachment of targeting ligands and poly(ethylene glycol) (PEG).

Additionally, factors like the membrane potential and the acidity of intracellular compartments play crucial roles in determining the stability of nanoparticles as well as final trafficking destination. As a result, pH‐responsive polymers have been investigated as delivery agents to overcome intracellular trafficking barriers. Intracellular pH can vary substantially by organelle. Endosomes and lysosomes typically exhibit pH values of 6.8–4.5.^[^ ^6^ ^]^ By judicious selection of materials and careful engineering of molecular architecture, nanomaterials can be developed to give well‐controlled pH response and drug release. Modifications to nanoparticles, such as conjugation with a targeting surface ligands or other functional molecules like chelators, significantly impact their performance. Modifications of these base properties have potential for targeting specific intracellular compartments and treating diseases associated with organelles’ dysfunction

Many considerations need to be made in the design of precision medicine.^[^ ^43^ ^]^ To target nanomaterials at the organelle level, researchers must consider physical, chemical, and biological properties, along with the needs of their target tissue and therapeutic goal. The physical properties of the material must be considered in conjunction with the delivery route and functionalization, as all these factors play a role in the interactions the particles face on their way to their desired target. To overcome this issue, various nanoparticle designs can be employed for subcellular localization. Nanoparticle characteristics—including morphology, size, shape, charge, and functionality—significantly affect the transport and delivery of therapeutics. Furthermore, nanomaterials can carry cargo meant to execute a specific role in the target. A range of therapeutics—including small molecules, nucleic acids, proteins, peptides, pharmaceutical drugs, reaction components/inhibitors, and even CRISPR systems for gene editing—can be delivered using these nanoparticles (Figure 2).^[^ ^43^, ^44^ ^]^ These conjugated molecules gain the benefit of more targeted delivery, potentially increasing therapeutic effect and decreasing dosage by limiting off‐target effects. Targeting the organelles can push this even further still.

## 4 Targeting the Organelles

### 4.1 Methods of Targeting Lysosomes/Endosomes

The endosomal‐lysosomal pathway is one of the most important mechanisms for nanoparticle internalization. This pathway modulates the uptake and degradation of cargo within the cell. Owing to their dimensions and uptake mechanisms, nanoparticles predominantly localize in endosomes, as direct membrane translocation is unlikely. These endosomes, originating from the endocytic transmembrane structures that mediate initial plasma membrane crossing, serve as the primary loci for nanoparticles. Independent of the mode of entry, endosomes are the initial intracellular compartments encountered by the nanoparticles.

**Figure 3:**
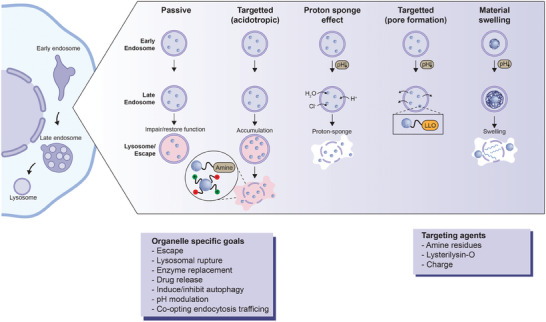
Targeting endosomes and lysosomes of the cell. Nanoparticles tend to follow the canonical endocytosis pathway ending in the lysosomal compartment. The endosomes and lysosomes can be the goal or barrier to other destinations. Nanoparticles can utilize both passive and specialized properties to escape this compartment. Passive properties allow for accumulation or swelling within endosomes and lysosomes causing nanoparticle release. Specialized properties can force nanoparticles to escape their endosomal compartment after entry, typically utilizing the pH shifts in the endosomal/lysosomal pathway leading to the acidotropic effect, proton sponge effect, pore formation, and swelling. The figure was created using icons from BioRender.com and modified and finalized in Adobe Illustrator.

Endosomes, characterized by its acidic milieu, temporally host the nanoparticles before their translocation or progression to the lysosomal degradation pathway. Within the endosomes, nanoparticles typically encounter a pH of 6.0 in the initial phase, decreasing to as low as 4.5 in the lysosomal transition stages.^[^ ^15^, ^45^ ^]^ Endocytosis plays a pivotal role in intracellular targeting of nanoparticles. Although numerous nanoparticles are engineered for rapid endosomal escape, for certain therapeutic applications, retention within the endosome and eventual transport to the lysosomal compartment is beneficial, facilitating compound degradation or activating therapeutic mechanisms through pH alteration. Different methodologies are employed to navigate these compartments, highlighting strategies that either co‐opt the endosomal‐lysosomal pathway or promote efficient endosomal escape (**Figure** **3**).

Early endosomes are characterized by an acidic pH, essential for the activity of functionality of endosome‐specific enzymes. This characteristic has been exploited for release of pH‐responsive nanoparticles into the cytosol. The inherent pH shift in the endosome serves as an efficient biological trigger in endosomal escape by nanoparticles. Most nanomaterials employ the “*proton sponge effect*”, where functional groups or slightly basic molecules sequester protons within the endosome/lysosome. This process intensifies proton pumping into the intracellular compartment, leading to an increased influx of ions and water.^[^ ^46^, ^47^ ^]^ This resulting osmotic swelling, ultimately causes endosomal rupture and release of the encapsulated cargo into the cytoplasm.^[^ ^45^, ^48^, ^49^ ^]^ Cationic polymers, which are able to electrostatically bind negatively charged nucleic acids such as DNA and RNA, promote endosomal rupture through this “proton sponge” mechanism. These polymers buffer incoming protons during endosomal acidification. This action causes an accumulation of protons and counter ions within the endosome, generating an osmotic pressure that leads to endosome rupture.

This approach has been utilized as a very efficacious method of delivery for small molecules including siRNA.^[^ ^50^ ^]^ Nanoparticles deficient in adequate surface charge or incapable of activating the proton sponge effect remain entrapped in the endosome. Other methodologies exist for nanomaterials to escape into the cytoplasm. Efficient endosomal escape with pH‐sensitive hydrophobic cores rapidly disassembles as the pH of endosomes decrease.^[^ ^51^ ^]^ This allows for the swelling and release of the incorporated therapeutic agents.

Well‐regulated endocytosis and endosomal trafficking are crucial for cellular growth and survival. Specifically, lysosomal/endosomal system is a key regulator of cellular homeostasis, involved in intra‐ and extracellular degradation, plasma membrane repair, cholesterol homeostasis, and apoptosis. Dysfunctional cargo trafficking is linked to Alzheimer's disease, Huntington's disease, and autism.^[^ ^2^, ^52^ ^]^ Whereas lysosome dysfunctions contribute to diseases such as cancer, neurodegenerative disorders, cardiovascular diseases, and storage disorders.^[^ ^6^, ^53^, ^54^ ^]^

These storage diseases, resulting from lysosomal hydrolase deficiency are characterized by abnormal lysosomal accumulation of un‐degraded substances. Therapeutically, inducing cell death through the pH‐responsive drug release in the lysosome is beneficial in cancer and delivering drugs or ions can address metabolic deficits in cells with impaired endosomal function. Lysosomal targeting is commonly used in cancer therapy to induce cell death.

Lysosomal storage disorders are involved in pathogenesis of neurodegenerative diseases. Strategies to enhance or restore lysosomal‐mediated degradation in cells by nanoparticles have been investigated. Poly(DL‐lactide‐*co*‐glycolide) (PLGA) nanoparticles can localize within the lysosome and have shown tremendous therapeutic promise. Due to their acidic nature, these PLGA nanoparticles traffic to and restore impaired lysosomal function in various cellular models including lysosomal‐related myopathy and Parkinson's Disease (PD).^[^ ^55^ ^]^ These nanoparticles are transported to the lysosome and ultimately lower lysosomal pH and restore degradative function, showing reduction in neurodegeneration in vivo.

Nanoparticles can accumulate in lysosomes over extended periods of time and can cause impairment of lysosomal function.^[^ ^56^, ^57^, ^58^, ^59^ ^]^ Silica (SiO_2_) nanoparticles have been shown to accumulate in the lysosome and block autophagy‐mediated protein turnover.^[^ ^56^ ^]^ Similarly, Copper oxide (CuO) nanoparticles taken by cells accumulated within lysosomes resulting in impairment of autophagy.^[^ ^57^ ^]^ This is attributed to the release of Cu ions from nanoparticle that result in toxicity due to oxidative stress and DNA damage. Moreover, Silver (Ag) and gold (Au) nanoparticles are also found to accumulate in lysosomal structures and have shown to influence lysosomal pH and impair their function.^[^ ^58^ ^]^ The natural accumulation of these nanoparticles in the lysosome impairs the normal lysosomal function and triggers cell death, which is one of the approaches to target and kill cancerous cells.

Additionally, in a more targeted approach, the bacterial pathogen *Listeria monocytogenes* has developed a strategy to secrete Listeriolysin O (LLO) toxin as a method to escape the eukaryotic lysosomal system upon infection; conjugation to the surface of gold nanoparticles similarly promotes their lysosomal escape.^[^ ^60^ ^]^ Endosomal acidification leads to release of the LLO protein from the nanoparticle surface and its self‐assembly into a pore that perforates the endosomal/lysosomal membrane, enabling the escape.

Exploiting lysosomal properties can guide modifications of nanoparticles to facilitate their accumulation in the lysosome. For instance, the acidic environment of lysosomes facilitates the enrichment of lipophilic amines, owing to their natural propensity to accumulate in acidic settings.^[^ ^61^ ^]^ This acidotropic effect has been leveraged in amine‐modified polystyrene nanoparticles to induce cancer cell death.^[^ ^62^ ^]^ Furthermore, emerging ultrasmall nanomaterials, such as organosilica nanodots and carbon dots measuring ∼2 nm and featuring amine groups, demonstrate inherent lysosome targeting capabilities. These have been successfully applied in advanced lysosomal imaging within cells.^[^ ^63^, ^64^ ^]^

Another approach involves pH‐dependent aggregation, where mixed‐charge nanoparticles with varying ratios of positively and negatively charged ligands specifically target lysosomes.^[^ ^47^ ^]^ This selectivity stems from pH‐dependent aggregation events, leading to lysosome swelling and subsequent disruption of lysosomal membrane integrity.^[^ ^65^ ^]^ This process impairs lysosomal functions, ultimately inducing cell death in cancer cells. Effective drug delivery has been demonstrated using nanocarriers and mixed‐charge nanoparticles, which enhance affinity for lysosomal membranes and promote cellular accumulation. This approach improves the efficiency of prodrugs and therapeutics in vivo, particularly in cancer models.^[^ ^66^, ^67^, ^68^ ^]^ Additionally, these nanocarriers have been exploited to reprogram immune cells by switching tumor‐associated macrophages from the M2 to the M1 phenotype for cancer immunotherapy. This reprogramming induces an adaptive immune response and leads to tumor regression in vivo.^[^ ^69^ ^]^

### 4.2 Targeting the Nucleus

The nucleus, as the primary organelle regulating reproduction, growth, metabolism, and the cell cycle, is central to therapies that focus on controlling nuclear‐governed processes and gene targeting. However, the double‐layered nuclear membrane, which isolates the nucleus from the cytoplasm, represents a significant barrier to nucleolar targeting due to its high selectivity in protecting the genetic material within. Nuclear targeting of nanomaterials typically involves drug and gene delivery processes. Most nanoparticles, used either as vehicles or for their general therapeutic effects, remain in the cytosol because the nuclear membrane impedes their entry.^[^ ^70^, ^71^ ^]^ Consequently, only a small percentage of the drugs delivered to the cytosol reaches the nucleus, significantly limiting the efficacy of treatments intended to act on nuclear components. Therefore, designing and preparing nanomaterials capable of translocating into the nucleus is crucial for enhancing the effectiveness of these therapies.

Potential applications for nuclear targeting include interventions on the nuclear envelope, alongside drug delivery and gene therapy. Defects in the nuclear envelope and its transport mechanisms have been associated with muscular dystrophy, as well as cardiac and skeletal myopathies, neurodegenerative diseases, and aging—conditions often linked to nuclear structural abnormalities.^[^ ^3^, ^72^, ^73^ ^]^ These defects typically stem from mutations in the nuclear lamina, a protein meshwork essential for maintaining the structural integrity of the inner nuclear membrane. Such mutations can lead to nuclear fragility and changes in nuclear positioning, gene expression, and subsequent impacts on the endoplasmic reticulum.^[^ ^72^ ^]^ Furthermore, many current anticancer drugs, which are DNA‐toxins that bind to nuclear DNA or associated enzymes, exert their cytotoxic effects on cancer cells. For these drugs to be effective, they must penetrate tumor cells and be delivered to the nucleus where they can perform their intended function.^[^ ^71^ ^]^

**Figure 4:**
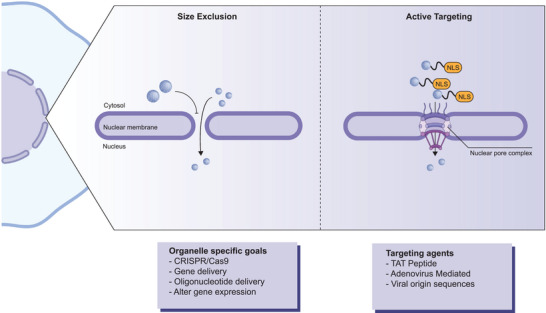
Targeting the nucleus. The nuclear envelope poses a highly critical barrier for the nuclear delivery of nanoparticles and their cargo. Size exclusion is a critical feature of this organelle. Nanoparticles and their cargo can obtain priority access and increased targeting ability via a nuclear localization sequence (NLS) through the nuclear pore complex (NPC). The figure was created using icons from BioRender.com and modified and finalized in Adobe Illustrator.

As gene therapy has evolved for the treatment of human disorders, it has been applied to diseases such as diabetes and cancers. This approach seeks to permanently treat or reverse genetic disorders by introducing novel DNA or repairing/replacing defective DNA. However, therapeutic genes face several extracellular and intracellular barriers. They must not only evade hydrolysis in the endosomes and lysosomes and traverse the cytosol but are also ultimately restricted by their ability to successfully pass through the nuclear envelope.^[^ ^74^, ^75^ ^]^ Due to these factors, the nucleus remains one of the most challenging organelles to target. Preserving nuclear integrity while delivering nanoparticles is therefore a primary obstacle in intracellular targeting (**Figure** **4**).

A primary factor to consider for nuclear targeting is nanoparticle size. The nuclear pore complex (NPC), which governs the permeability of the membrane, permits the passage of mRNA, pre‐ribosomal proteins, and other essential cargo of very limited size.^[^ ^76^ ^]^ The capacity of a nanoparticle to traverse the nuclear envelope depends on its ability to fit within the channels that span the envelope. Studies on the size‐dependent penetration of gold nanoparticles revealed that particles smaller than 10 nm (specifically, 2 and 6 nm) could enter the nucleus, while larger ones (10 and 16 nm) remained localized to the cytoplasm.^[^ ^77^ ^]^ In this study, 2 nm particles successfully carried a triplex‐forming oligonucleotide (TFO) into the nucleus, where it bound to a promoter and altered gene expression more effectively than the agent alone.^[^ ^77^ ^]^

The transport of larger heterogeneous nanoparticles can be enhanced using a nuclear localization sequence (NLS), often derived from viruses. This approach is widely utilized, with NLS sequences shown to effectively target nuclei through conjugation to various types of functionalized nanoparticles, such as silver, quantum dots, and magnetic nanoparticles. NLS conjugation facilitates the binding of import receptors importin α and β (karyopherin) in the cytoplasm, subsequently enabling nuclear translocation.^[^ ^74^, ^75^, ^78^, ^79^, ^80^ ^]^ The TAT peptide, an 11‐amino‐acid sequence from the HIV‐1 transactivating protein, is one of the most commonly used and versatile NLS peptides for aiding the localization of nanoparticles to the nucleus.^[^ ^28^, ^81^, ^82^ ^]^ in vivo, the TAT peptide can improve efficacy of photodynamic and photothermal therapy treatments by improving specificity and localization of even larger 100 nm particles in cancer cells for effective destruction of genetic material.^[^ ^83^ ^]^

This peptide has been used to transport mesoporous silica nanoparticles of various sizes into the nucleus for the delivery of anticancer drugs.^[^ ^84^ ^]^ Studies demonstrate that particles smaller than 50 nm can efficiently target and deliver therapeutic agents to the nucleus.^[^ ^85^ ^]^ Furthermore, NLS sequences from other sources, such as those derived from the simian virus SV40, bipartite nucleoplasmin, and adenoviruses, have been proven effective in transporting various nanoparticles of differing sizes to the nucleus.^[^ ^86^, ^87^ ^]^ This versatility underscores the potential of NLS‐modified nanoparticles as a robust strategy for enhancing nuclear drug delivery.

### 4.3 Targeting Mitochondria

Mitochondria are the primary sites for ATP generation in cells and perform essential metabolic functions. Therefore, monitoring, modulating, and protecting mitochondrial function is crucial for normal cellular operations. Mitochondrial dysfunction is linked to a variety of human diseases, including cancer, gastrointestinal disorders, cardiac and respiratory failure, neurodegenerative and neuromuscular diseases, obesity, and diabetes.^[^ ^1^, ^88^, ^89^, ^90^, ^91^, ^92^, ^93^, ^94^, ^95^, ^96^ ^]^ Mitochondrial diseases can arise from environmental and genetic factors that affect mitochondrial DNA (mtDNA), as well as issues in mitochondrial structural proteins, defective mitochondrial RNA synthesis, and respiratory chain deficiencies. The clinical manifestations of mitochondrial diseases involve dysfunction across various organs and tissues.

Specifically, the rapid proliferation of cancer cells significantly increases the energy demands on mitochondria, leading to altered states of glucose, lipid, and amino acid metabolism, along with elevated levels of hexokinases.^[^ ^95^, ^97^, ^98^ ^]^ Common in endocrine disorders like diabetes mellitus are mutations in mitochondrial protein synthesis genes. Additionally, sporadic large‐scale mutations may occur, resulting in cardiac abnormalities, deafness, weakness, and dementia.^[^ ^47^ ^]^ Ocular diseases such as Leber's hereditary optic neuropathy stem from known mtDNA point mutations, while cataracts, glaucoma, and macular disease are associated with mitochondrial damage.^[^ ^91^, ^96^, ^99^, ^100^ ^]^ In neurodegenerative conditions like Parkinson's and Alzheimer's disease, cells exhibit altered glucosidase activity, mitochondrial stress, and diminished glucose uptake.^[^ ^101^ ^]^

Mitochondria are semiautonomous organelles composed of inner and outer membranes, which pose specific challenges for localizing therapeutics within the mitochondrial matrix. Molecule transport into mitochondria relies on the mitochondrial membrane potential and the organelle's import machinery.^[^ ^53^ ^]^ By exploiting the inherent size, shape, and charge of various nanoparticles, we can target these therapeutics to the mitochondrion. The outer mitochondrial membrane (OMM) serves as a barrier between the mitochondrion and the cytoplasm. The voltage‐dependent anion channel (VDAC) is the sole ionic channel facilitating rapid exchange of molecules and ions between these compartments.^[^ ^102^ ^]^

**Figure 5:**
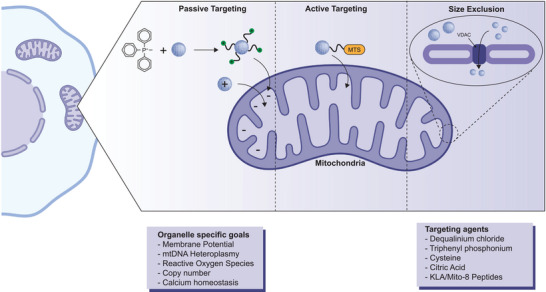
Targeting the mitochondrion. A semiautonomous organelle, the mitochondria is an important and frequently targeted organelle. The mitochondrial membrane, like outer and nuclear membrane, poses a physical barrier to entry of large objects. Positively charged molecules, such as lipophilic cations, can passively deliver cargo by exploiting the high membrane potential of mitochondria (the inner mitochondrial matrix is negative with respect to the intermembrane space). Recognition of N‐terminal mitochondrial targeting sequences (MTSs) engage receptors on the outer membrane and the translocation machinery. Nanoparticles displaying MTS can enter into mitochondria through this process. The figure was created using icons from BioRender.com and modified and finalized in Adobe Illustrator.

Due to the selectivity of the mitochondrial membrane, nanomaterials aiming to enter this organelle require the intentional design of several factors to promote entry (**Figure** **5**). Understanding the permeability of this barrier and strategically navigating nanoparticles across it without causing unintended damage is crucial, particularly where mitochondrial function can be restored. Inappropriate force during nanoparticle entry can compromise the mitochondrial membrane, potentially disrupting the proton motive force and triggering apoptosis. Studies have demonstrated that while 6nm gold nanoparticles cannot traverse the OMM, 3 nm particles can pass through via VDAC.^[^ ^102^ ^]^ This size‐selective behavior has also been confirmed for other metal nanoparticles and quantum dots smaller than 10 nm.^[^ ^103^ ^]^

Apart from nanoparticle size, the mitochondrial membrane potential (ΔΨm) is a critical factor in the development of targeted nanoparticles. Leveraging cationic compounds enables nanoparticle accumulation in the mitochondrial matrix via electrostatic interactions. ΔΨm, maintained at 150–180 mV lower than the cytosol, is pivotal for the organelle's biological functions. It facilitates the influx of protons and Ca^2+^ into mitochondria and influences O_2_ generation. For instance, liposome‐like vesicles incorporating the lipophilic cation Dequalinium chloride exhibit high affinity for the mitochondrial membranes of carcinoma cells.^[^ ^104^, ^105^ ^]^ In this way, cargo coated with specific charges has been effectively used in cancer treatment to induce mitochondrial apoptosis pathways.^[^ ^67^ ^]^

Triphenyl phosphonium (TPP) is the most frequently used and well‐developed cation for mitochondrial targeting due to its lipophilic and hydrophilic properties that facilitate the localization of dequalinium chloride and TPP, respectively.^[^ ^106^, ^107^ ^]^ The positively charged cationic phosphonium and triphenyl group are unique to TPP, which has a low energy requirement for entry into the mitochondrial membrane, enhancing its passage through the membrane.^[^ ^67^, ^108^, ^109^, ^110^, ^111^ ^]^ Gold nanoparticles and end‐functionalized PLGA‐PEG nanoparticles utilizing this cation have been demonstrated to effectively localize to mitochondria, facilitating drug delivery.^[^ ^97^, ^112^ ^]^

Biodegradable silica nanoparticles modified with TPP and cell‐penetrating poly‐disulfides facilitate rapid intracellular uptake and mitochondrial localization.^[^ ^113^ ^]^ TPP‐modified micelles and gold nanoparticles decorated with 3‐bromopyruvate (a hexokinase inhibitor) target the elevated mitochondrial membrane potential in cancer cells, disrupting glycolysis and altering mitochondrial bioenergetics.^[^ ^67^ ^]^ Additionally, TPP‐targeted ceria nanoparticles act as ROS scavengers, mitigating neuronal death in Alzheimer's disease models by scavenging reactive oxygen and nitrogen species and reducing mitochondrial morphological damage.^[^ ^111^, ^114^ ^]^

Furthermore, TPP‐conjugated molybdenum disulfide quantum dots (TPP‐MoS_2_ QD) protect against Alzheimer's by targeting mitochondria, mitigating Aβ aggregate‐mediated neurotoxicity, and eliminating Aβ aggregates.^[^ ^115^ ^]^ Rhodamine, another widely used lipophilic cation, accumulates inside mitochondria and is utilized in mitochondrial probes for microscopy.^[^ ^116^, ^117^ ^]^ Surface modifications like a cysteine/citric acid coating on gold nanoparticles enhance mitochondrial localization due to the organelle's role in cysteine oxidation reactions.^[^ ^116^, ^117^ ^]^

Further supporting TPP's versatility and extensive characterization, in vivo applications of TPP‐based strategies have proven fruitful across multiple therapeutic areas. Modifications to TPP have been successful in enhancing its effectiveness. For instance, mitochondria‐targeted (3‐carboxypropyl) triphenylphosphonium bromide (CTPP) and triphenylphosphonium‐conjugated pyropheophorbide‐a (TPPa) have been developed to address limitations in photodynamic therapy, which aims to generate ROS in target tissues but is known to affect multiple targets nonspecifically.^[^ ^118^, ^119^ ^]^ By localizing to the mitochondria, these particles improve treatment efficacy by overcoming tumor hypoxia and selectively targeting cancer cells. In Alzheimer's disease research, TPP has been utilized to guide quantum dots to switch microglia to an anti‐inflammatory phenotype, thereby preventing neuroinflammation. Additionally, TPP has been incorporated into micelles, ceria nanoparticles, and lipid nanoparticles to reverse mitochondrial dysfunction through the delivery of antioxidants—with micelles effectively restoring cognitive performance in transgenic mice.^[^ ^114^, ^115^, ^120^, ^121^ ^]^ TPP has undergone extensive validation across a variety of applications, demonstrating its high specificity and efficiency in therapeutically targeting mitochondria. This extensive validation makes TPP‐based strategies among the most promising for clinical use.

Other strategies for mitochondrial targeting include the use of mitochondria‐targeting peptides or mitochondrial targeting sequences (MTS). These sequences are typically located at the N‐terminal of proteins and share physicochemical properties recognized by the mitochondrial import machinery. By incorporating lipophilic residues and positively charged amino acids such as arginine and lysine, these peptides facilitate mitochondrial localization. For instance, the peptide‐based mitochondrial targeting sequence Mito‐8 has been conjugated to the surface of quantum dots (QDs).^[^ ^122^ ^]^ Additionally, the KLA peptide (D[KLAKLAK]2), originally developed as an antimicrobial peptide to selectively disrupt bacterial membranes, has also been shown to specifically target and compromise mitochondrial membranes in eukaryotic cells.^[^ ^123^ ^]^ Once internalized, KLA peptide localizes to the mitochondria, disrupting their membranes to initiate cell apoptosis.^[^ ^98^, ^124^, ^125^ ^]^ On 13.5 nm gold nanoparticles (AuNPs), this peptide targeted HeLa cell mitochondria, inducing swelling, loss of membrane potential, and ultimate cell death, proving more toxic than either non‐functionalized counterparts or the peptide alone.^[^ ^124^ ^]^

### 4.4 Targeting Endoplasmic Reticulum

The Endoplasmic Reticulum (ER) is the largest organelle in the cell and serves as a critical hub for protein synthesis, transport, and metabolism. It plays a key role in exporting both soluble molecules and membrane‐bound substances. Additionally, the ER is integral to various signaling pathways and cellular stress responses, including intracellular calcium homeostasis, protein secretion, and lipid biosynthesis.^[^ ^5^, ^126^ ^]^ Located adjacent to the cell nucleus, the ER is studded with ribosomes that specialize in synthesizing proteins from mRNA. These proteins, equipped with signal sequences, are directed to the organelle. While some proteins remain within the ER, others are transported to the Golgi apparatus via the ER‐Golgi pathway for further processing before being directed to the plasma membrane or secreted externally. Moreover, the ER serves as a major reservoir of Calcium (Ca), which is used as a pervasive signaling molecule that can influence protein dynamics within the cell. This multifunctional role underscores the ER's significance in maintaining cellular function and coordinating complex biochemical processes.^[^ ^127^ ^]^

Given its close proximity to the nucleus, the Endoplasmic Reticulum (ER) can swiftly relay signals regarding issues in protein synthesis, translocation, and folding. Disruptions in homeostasis and the accumulation of misfolded proteins can trigger an ER stress response. Prolonged ER stress is known to lead to apoptosis and has been implicated in a range of conditions, including cardiovascular diseases, diabetes, neurodegenerative diseases, insulin resistance, and certain cancers.^[^ ^126^, ^128^, ^129^, ^130^, ^131^, ^132^, ^133^ ^]^ Of particular concern in many diseases is the irregular functioning of the ER because of the unfolded protein response (UPR). Various cellular disturbances can lead to the accumulation of unfolded proteins in the ER, activating this evolutionarily conserved response. Conditions such as stroke, ischemia‐reperfusion injury, and various heart diseases induce hypoxia and hypoglycemia, which in turn cause protein misfolding and subsequent ER stress through mechanisms involving nitric oxide and reactive oxygen species.^[^ ^134^ ^]^ This ER stress response subsequently halts protein synthesis, promotes the refolding of proteins, and facilitates the clearance of misfolded proteins, crucial steps in restoring cellular function and preventing further damage.

The ER directly interacts with and works tightly with the endocytosis pathway.^[^ ^135^, ^136^, ^137^, ^138^ ^]^ Macromolecules can be transported directly to the ER, effectively bypassing the lysosomal pathway.^[^ ^25^ ^]^ This method represents a promising route for targeting particles to the ER. For specific ER targeting in cells, leveraging the interaction between ER‐specific enzymes and their substrates is critical. Grafting ER‐targeting ligands onto small molecules and nanoparticles can facilitate their efficient accumulation in the ER.

Given the variety of pathologies resulting from ER stress, nanoparticles localized to the ER have significant potential to modulate stress in cells of interest. This approach is particularly valuable in cancer treatment, where inducing stress can trigger cell death while sparing surrounding healthy tissue. Examples of such ER‐targeting ligands include small molecules like the p‐toluenesulfonyl (Tosyl) group and peptides such as the KDEL sequence.^[^ ^139^, ^140^, ^141^ ^]^ These ligands can recognize and bind to site‐specific receptors or ER components, enhancing the ER‐targeting capabilities of NPs. The sulfonamide moiety, a member of the Tosyl family, is particularly noted for its high selectivity and affinity for the ER and has been widely used in ER‐targeting probes and drugs.^[^ ^123^, ^139^ ^]^ Lipid nanoparticles, for example, have been demonstrated to induce ER stress specifically in cancer cells.^[^ ^123^, ^139^ ^]^ They localize through the ability of prolonged ER stress to activate apoptotic pathways makes these nanoparticles an invaluable tool in oncology. By impairing multiple targets within the ER, lipid nanoparticles can serve as a primary treatment method or as a platform to enhance the efficacy of new or existing therapeutic modalities. This dual functionality underscores the potential of nanoparticle‐based strategies in treating a range of cancers by leveraging the cellular mechanisms of ER stress.

Similarly, another sulfonyl ligand, has been conjugated to graphene oxide nanoparticles for targeted ER localization and subsequent drug delivery.^[^ ^142^ ^]^ The KDEL peptide, consisting of the amino acid sequence Lys‐Asp‐Glu‐Leu, represents another common ER‐targeting strategy. In physiological contexts, protein trafficking to the ER is mediated by the KDEL sequence interacting with its receptor in the ER‐Golgi intermediate compartment; this interaction is critical for the improved intracellular targeting of these organelles. KDEL peptide is recognized by a specific receptor and transported back to the ER via a coat protein I (COPI) mechanism. Studies have shown that gold NP‐KDEL nanoconjugates were internalized by cells through clathrin‐mediated endocytosis and subsequently transported from the Golgi apparatus to the ER, thereby avoiding lysosomal degradation. This strategic use of specific peptides and ligands demonstrates the potential of precision targeting within cellular substructures, enhancing the efficacy of therapeutic interventions.^[^ ^140^ ^]^ These featured characteristics of the KDEL peptide make it an attractive and favored tool for ER retention of therapeutic agents.

Nanoparticles camouflaged with specific membrane components can exploit the natural functions of the ER, tricking cells into transporting these particles intracellularly. This strategy utilizes small target fusion proteins (t‐SNAREs) located on the surface of biological membranes and their counterparts in the ER (SNAREs). Nanoparticles disguised with cancer cell membranes demonstrate enhanced biological clearance and are preferentially transported to the ER, where these proteins play a crucial role in intracellular trafficking.^[^ ^143^, ^144^ ^]^ This had been effectively used in homologous cancer cell membrane‐coated nanoparticles, used to target the ER and sequentially engage and block ER pathways through Brefeldin and COPI, reversing immunosuppression and promoting anti‐tumor immunity.^[^ ^145^ ^]^

This approach has been effectively employed with mesoporous silica nanorods and cell‐membrane‐coated PLGA nanoparticles to target the ER in cancer models.^[^ ^145^, ^146^ ^]^ Additionally, a novel synthesized targeting peptide, Penetratin, has shown promise in aiding the localization of lipid/polymer hybrid nanoparticles to the ER. Penetratin contains short cationic peptides, including lysine, arginine, and proline, which are preferentially recognized by ER transport proteins. This recognition facilitates the successful targeting of these nanoparticles to the ER, demonstrating the potential of these engineered particles in precise intracellular delivery and therapeutic applications.^[^ ^147^ ^]^

### 4.5 Targeting Golgi Apparatus

**Figure 6:**
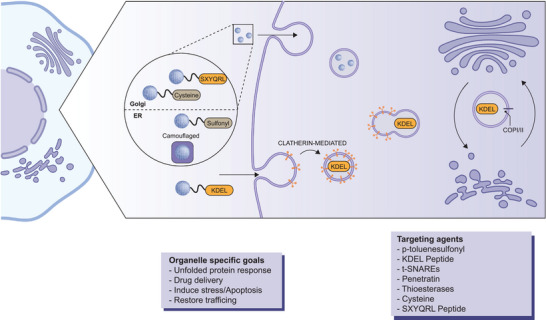
Targeting of the Endoplasmic reticulum and Golgi apparatus. Membrane camouflaged and particles with sulfonyl ligands can mediate ER delivery, receptors on the ER respond to sulfonyl on nanoparticle surface. Specific Golgi targeting sequences and cysteine‐rich ligands mediate Golgi targeting through mimicry of the binding sites of Golgi proteins. Additionally, retrograde trafficking of proteins from the Golgi to the endoplasmic reticulum (ER) is enabled by the KDEL sequence, which is recognized and transported by the KDEL receptor, trafficking via coat protein complex I (COPI) and coat protein complex II (COPII). The figure was created using icons from BioRender.com and modified and finalized in Adobe Illustrator.

The Golgi apparatus serves as a critical processing and sorting hub within each cell, primarily focused on two main functions: post‐translational modification of proteins and the sorting, packaging, routing, and recycling of these modified cargoes to their appropriate cellular destinations. This organelle plays a key role in handling membrane and secretory proteins, which are essential for virtually every aspect of cellular function. The Golgi apparatus processes a diverse array of biological molecules, including secretory proteins, glycoproteins, cell membrane proteins, lysosomal proteins, and glycolipids, all of which are crucial for maintaining proper cellular operations and functionality. Proteins synthesized and modified in the endoplasmic reticulum (ER) are incorporated into ER‐derived carriers and transported to the Golgi apparatus. There, they undergo further modifications and are meticulously sorted for delivery to their final destinations. In this way, the ER and Golgi work closely together, and many targeting strategies involve one or both of these organelles and the transport between them (**Figure** **6**).^[^ ^148^, ^149^ ^]^

The nervous system, skin, bone, cartilage, and skeletal muscle are particularly sensitive to changes in intracellular trafficking, with mutations in conserved Golgi genes affecting essential functions leading to a range of disorders. These can manifest as wrinkled skin, neurodegenerative diseases, muscular dystrophy, and skeletal dysplasia.^[^ ^130^, ^150^, ^151^ ^]^ In the nervous system, the functionality of intracellular trafficking critically influences neuronal development, homeostasis, and neurodegeneration. Neurons, being highly polarized cells, require extensive intracellular trafficking to facilitate the growth of dendrites and axons, which significantly expands the cell surface area. Similarly, skin, bone, cartilage, and skeletal muscle are composed largely of extracellular matrix (ECM), which defines their structure and physical properties. Nearly all ECM components depend on intracellular trafficking systems for transport. Any alterations in Golgi apparatus membrane trafficking can lead to glycosylation abnormalities, crucially affecting the assembly and maintenance of the ECM. Such impairments in matrix protein glycosylation can significantly disrupt the structural integrity and function of these tissues.^[^ ^151^ ^]^

Many clinical conditions are linked to dysfunctions or mutations in Golgi apparatus proteins. For instance, GOLPH3, a member of the trans‐Golgi network protein family, is significantly overexpressed in glioma tissues, with its expression level correlating directly with tumor malignancy.^[^ ^152^ ^]^ GOLPH3 also plays a role in the Golgi stress response, where stress‐induced upregulation of this gene leads to Golgi fragmentation and apoptosis, a process observed in cerebral ischemia‐reperfusion injury—an important pathological mechanism in ischemic stroke.

Moreover, mutations such as TRAPPC2, which affect the membrane trafficking pathway between the ER and Golgi in bone cells and chondrocytes, result in X‐linked spondyloepiphyseal dysplasia. This condition exemplifies how disruptions in Golgi function can lead to significant skeletal abnormalities. Additionally, compromised Golgi function that affects trafficking and apoptosis pathways can contribute to the progressive neuronal loss observed in Parkinson's disease. These examples underscore the critical role of Golgi apparatus proteins in maintaining cellular function and integrity across various tissues and systems, highlighting their importance in understanding and treating a wide range of diseases.^[^ ^151^ ^]^

Macromolecules undergoing caveoli‐mediated endocytosis typically find their path directed toward the Golgi apparatus or endoplasmic reticulum. Enhancing the specificity of targeting the Golgi can ensure a higher efficiency of particle delivery to this crucial organelle. This capability is particularly valuable in the delivery of drugs to cancer cells and the transport of metabolites to and from the membranes. To facilitate this localization, ligands that mimic the binding sites of natural Golgi‐targeting proteins can be employed.^[^ ^153^ ^]^ For instance, types of peptide thioesters, which are substrates for thioesterases, enter cells and are hydrolyzed by Golgi‐associated thioesterases. The resulting thiopeptides then form dimers that accumulate in the Golgi, thus enhancing the concentration of therapeutic agents where they are most needed. Additionally, proteins such as galactosyltransferase (a type of glycosyltransferase) and protein kinase D, known for their rich cysteine residues, naturally localize to the Golgi apparatus. These proteins play essential roles in the processing of metabolites and proteins as they transit through the organelle.^[^ ^154^, ^155^, ^156^ ^]^ Exploiting the cysteine‐rich nature of these enzymes, researchers have synthesized and functionalized carbon quantum dots and silica nanoparticles with abundant L‐cysteine residues. These nanoparticles have shown excellent long‐term localization to the Golgi, having been transported there from late endosomes. This targeted delivery system is not only effective for fluorescent imaging but also for precise drug delivery, demonstrating the potential for enhancing therapeutic efficacy through advanced nanoparticle engineering.^[^ ^157^ ^]^

Another innovative functionalization approach utilizes biocompatible, non‐immunogenic chondroitin sulfate polysaccharide.^[^ ^158^ ^]^ This polysaccharide facilitates Golgi apparatus targeting through interactions with N‐acetylgalactosaminyltransferases, enzymes that specifically recognize and transfer N‐acetylgalactosamine to polypeptide chains within the Golgi. Given that N‐acetylgalactosamine is a primary component of chondroitin sulfate, it effectively aids in the localization of nanomicelles and lipid nanoparticles to the Golgi in various cancer cell types.^[^ ^159^, ^160^, ^161^ ^]^ Effective in vivo suppression in tumor growth had been observed through disruption in Golgi structure with retinoic acid conjugated chondroitin sulfate.^[^ ^160^ ^]^ Similarly, chondroitin sulfate lipid nanoparticles loaded with doxorubicin and retinoic acid improved antitumor efficacy.^[^ ^159^ ^]^

Additionally, specific peptides have been identified that enhance targeting of the Golgi apparatus. For example, the peptide sequence SXYQRL promotes Golgi localization by initiating retrograde transport from endosomes to the Golgi. This peptide has been effectively used to selectively deliver therapeutic agents, such as retinoic acid, directly to the Golgi apparatus. In conjunction with platelet‐camouflaged nanoparticles, this targeted delivery system, incorporating retinoic acid, has been employed to mitigate damage caused by defective proteins in conditions like rheumatoid arthritis.^[^ ^162^ ^]^

In summary, targeting the Golgi apparatus remains a highly effective strategy for addressing various pathologies, due to its central role as a hub of processing and transport within the cell. The techniques described here have been successfully used to deliver nanoparticles and their associated therapeutic cargoes, aiming to prevent disease progression and positively influence cell fate.

## 5 Conclusions and Perspective

This review highlights the critical role of organelles in the burgeoning field of precision medicine, emphasizing how nanomaterials targeted to these cellular components can revolutionize therapeutic approaches. Organelles, as the smallest units of shared functions within cells, are pivotal in the development of treatments for a wide array of diseases, including diabetes, cancers, and neurodegenerative disorders. Advanced treatment of diseases will require vehicles that can deliver their payload in a highly regulated and site‐specific manner to achieve therapeutically relevant concentrations in subcellular organelles. The use of nanomaterials offers a unique opportunity due to their high surface area, chemical versatility, and ability to be functionalized with bioactive molecules, enhancing therapeutic efficacy and specificity. This work not only summarizes the unique roles and functions of eukaryotic cell organelles but also illustrates current advancements in tailoring nanomaterials for organelle‐targeted therapies. The potential for these innovations to improve health outcomes and foster the development of novel treatment strategies is profound, marking a significant step forward in the evolution of patient‐specific therapeutics.

While the controlled delivery at the organelle level has been achieved, their adaption to current medical practice have yet to be fully exploited. Mitochondria and nuclear transport are popularly validated targets in nanomaterial‐facilitated organelle‐targeted therapy in vitro and vivo because of their vital importance in cellular metabolism and in determining cell survival or death. Delivery of nanomaterials to intercellular compartments in vivo remains a complex process, and the molecules TPP and TAT have been extensively explored in vivo for targeting the mitochondria and nucleus, respectively. The translation of lab‐scale findings to effective therapeutics that have been verified in vivo has been slow but productive. In the context of therapeutic applications, the utilization of systemically administered drugs and nanoparticles alike face the same limitations. Rapid clearance, off‐target effects, and inefficient delivery affect therapeutic potential. In reality, a combination of approaches is necessary, where researchers combine targeting of affected tissues, like those that utilize unique pathological biology, in addition to their organelle targeting.

Even so, the field of advanced therapeutics with high specificity to targets is ever advancing. Artificial intelligence (AI) and computational models have furthered the potential design considerations. The heterogeneity between patients and pathologies of the same type make design difficult. Integration of AI can assist researchers in considerations of all the physical properties and factors discussed in this review that make organelle targeting possible. The nanoparticles discussed in this review have gone through many challenges in validation and have yet further to go for successful validation clinically. AI and machine learning can bridge the gap in design, making considerations of nanomaterial properties and their effects on transport and localization easier to rationalize and improved. Several models have already been utilized for predicting the ability of nanoparticles to permeate across the blood‐brain barrier and their potential toxicity.^[^ ^163^, ^164^, ^165^ ^]^ Extensive physical, chemical, and biological knowledge loaded into computational models can help make the design and study of nanomaterials less cumbersome for researchers aiming to address specific pathologies.

With many of these organelle targeting strategies demonstrated both in vitro and in vivo, their use will surely increase with time. It will be imperative to address the regulatory landscape behind these strategies as they develop in the near future. While passive targeting has therapeutic potential, it is limited by the heterogeneity of physiology. Despite extensive pre‐clinical studies, only a handful of passively targeted nanomaterials have been advanced beyond. The major factor limiting this has been determined to be unclear and mismatched guidelines. Many countries maintain different regulations concerning these materials with different definitions and classifications that do not align; even so, prolonged multi‐stage testing in animals is prolonged and costly but necessary. This, along with differing scrutiny around analytical methods, in vitro and physiochemical characteristics, and lacking in vivo translation have proven to limit targeted nanomaterial's widespread adoption and approval.^[^ ^166^, ^167^ ^]^ These factors have caused targeted nanotherapies to be unable to find appropriate commercial partners due to the challenging gaps in translation through these regulatory hurdles. While the controlled delivery at the organelle level has been achieved, their adaption to current medical practice have yet to be fully exploited. Multidisciplinary teams must work closely with the regulatory agencies to aid in the development of science‐based standards to shape current regulatory frameworks.

Ultimately, the most translatable potential are with those that show in vivo replication of in vitro data, an aspect that is lacking in this emerging field at this time. Further studies into these nanoparticles are essential to fully understand their potential limitations, particularly through rigorous in vivo testing. Immunogenicity and biocompatibility must be confirmed at the organismal level. While in vitro models have been useful in the screening and development of these promising therapeutics, they cannot fully replicate the complexity of the organism. Increased in vivo disease models will be necessary to push the generation of data to aid in the adoption of this potentially groundbreaking therapeutic. This is essential to advance from the lab to clinical applications, providing necessary insights to refine nanoparticle design, minimize risk, and address regulatory concerns. The efficiency supported by increasing targeting beyond tissues to the underlying organelle of the cell promises advancements in treatment strategies yet to be unlocked.

## Conflict of Interest

The authors declare no conflict of interest.
